# Update on the role of melatonin in the prevention of cancer tumorigenesis and in the management of cancer correlates, such as sleep-wake and mood disturbances: review and remarks

**DOI:** 10.1007/s40520-013-0118-6

**Published:** 2013-09-18

**Authors:** Mariangela Rondanelli, Milena Anna Faliva, Simone Perna, Neldo Antoniello

**Affiliations:** 1 Section of Human Nutrition, Health Sciences Department, Azienda di Servizi alla Persona, University of Pavia, Pavia, Italy; 2 Medical Direction, RSA F. Pertusati, Azienda di Servizi alla Persona, Pavia, Italy; 3 Endocrinology and Nutrition Unit, Section of Human Nutrition and Dietetics, Department of Applied Health Sciences, Faculty of Medicine, ASP, University of Pavia, Pavia, Italy

**Keywords:** Cancer, Melatonin, Mood, Sleep

## Abstract

The aim of this article was to perform a systematic review on the role of melatonin in the prevention of cancer tumorigenesis—in vivo and in vitro—as well as in the management of cancer correlates, such as sleep-wake and mood disturbances. The International Agency for Research on Cancer recently classified “shift-work that involves circadian disruption” as “probably carcinogenic to humans” (Group 2A) based on “limited evidence in humans for the carcinogenicity of shift-work that involves night-work”, and “sufficient evidence in experimental animals for the carcinogenicity of light during the daily dark period (biological night)”. The clinical implications and the potential uses of melatonin in terms of biologic clock influence (e.g. sleep and mood), immune function, cancer initiation and growth, as well as the correlation between melatonin levels and cancer risk, are hereinafter recorded and summarized. Additionally, this paper includes a description of the newly discovered effects that melatonin has on the management of sleep-wake and mood disturbances as well as with regard to cancer patients’ life quality. In cancer patients depression and insomnia are frequent and serious comorbid conditions which definitely require a special attention. The data presented in this review encourage the performance of new clinical trials to investigate the possible use of melatonin in cancer patients suffering from sleep-wake and mood disturbances, also considering that melatonin registered a low toxicity in cancer patients.

## Methods

In this article we aim at systematically reviewing the state of the art on the role of melatonin in the prevention of cancer tumorigenesis—in vivo and in vitro—as well as in the management of cancer correlates, such as sleep-wake and mood disturbances. In terms of search strategy, English written articles were sourced in September 2012 by means of PubMed (no date restriction) using the keywords hereinafter specified at the beginning of each chapter. Moreover, reasons and criteria based on which the reported scientific articles have been selected are also specified.

### The circadian hormone melatonin and its main function

This research has been carried out based on the keywords: “melatonin” AND “function” AND “circadian”; 5,608 articles were sourced. Among them, classical references have been selected and discussed.

Melatonin (*N*-acetyl-5-methoxytryptamine) is a hormone produced at night in the corpus pineale according to a rhythmical pattern and is controlled by an endogenous clock located in the hypothalamic suprachiasmatic nucleus [1].

Although it is primarily produced by the pineal gland, a number of other areas, such as the gut, retina, skin, and leukocytes [2, 3] also produce Melatonin.

Its main function is to synchronize the circadian rhythm, which means the pineal hormone melatonin (*N*-acetyl-5-methoxytryptamine) actually acts as a neuroendocrine transducer of the light–dark cycle [1].

Thanks to its action, melatonin has been tested as a treatment for a wide range of sleep disorders, including jet lag, shift-work, primary insomnia, sleep-wake cycle disruption as well as sleep problems in both children and elderly suffering from neurodevelopmental disorders [4–6].

The relationship between melatonin and sleep was initially investigated after registering in humans a higher level of circulating melatonin at night; furthermore, it was also noticed that the highest urinary 6-sulphatoxymelatonin excretion was actually coinciding with the highest nocturnal sleepiness [7]. In addition it has to be mentioned that, at physiologic doses, melatonin also induces sleep onset and maintenance, decreases sleep latency, improves sleep efficiency and overall increases the total sleep time [8, 9].

Melatonin seems also to be successful in the treatment of some aging-associated processes, such as disturbances of sleep/wake rhythm, since the endogenous production of indoleamine actually decreases with age.

Melatonin is mostly known as a circadian hormone, although it also prescribed as its sedative [10, 11], anxiolytic [10, 11], analgesic [12, 13], antihypertensive [14, 15], non-inflammatory [16], and oncostatic effects [17–19].

In particular, melatonin seems to produce a possible antidepressant effect [20, 21]—probably due to the action it performs on the central circadian regulation [22]—and also to improve cognitive functions [23, 24].

Disturbances in the rhythm and amplitude of melatonin secretion may account for symptomatic disturbances to sleep and mood. Moreover, the close association between sleep and mood disorders also suggests that melatonin may play an important role in mood management [25, 26].

Melatonin treatment not only improved total sleep time, but also reduced depressive symptoms [20, 26–28], thus indicating a correlation between sleep disturbance and depression.

### Melatonin secretion in cancer patients: what happens?

This research has been carried out based on the keywords: “melatonin” AND “secretion” AND “cancer”; 248 articles were sourced. Among them, the most significant epidemiological and retrospective, case–control studies have been selected and discussed.

In patients suffering from breast, endometrial, or colorectal cancer [29] melatonin secretion is impaired. The increased incidence of breast and colorectal cancer observed in nurses and other night-shift workers suggests a possible correlation between the reduced melatonin secretion and their increased light exposure at night [30, 31]. The physiological surge of melatonin at night is thus considered a “natural restraint” on tumor initiation, promotion, and progression.

The International Agency for Research on Cancer (IARC) recently classified “shift work that involves circadian disruption” as “probably carcinogenic to humans” (Group 2A) on the basis of “limited evidence in humans for the carcinogenicity of shift-work that involves night work”, and “sufficient evidence in experimental animals for the carcinogenicity of light during the daily dark period (biological night)” [32].

The epidemiologic evidence of a relationship between shift and night work and breast cancer in women is based upon nine different studies, six of which suggest a moderately increased risk to develop breast cancer after prolonged exposure to shift and night work [33]. The possible physio-pathological mechanisms actually relate to the internal disruption of biological circadian rhythms and clock genes, melatonin suppression through light by night, sleep deprivation.

Among the above-mentioned retrospective case–control studies, several of them registered in the blood and/or urine of patients suffering from breast cancer a lower melatonin or melatonin metabolite concentrations compared with breast cancer–free women [34–37]. In particular, a study [36] on the correlation between main melatonin metabolite excretion and plasma melatonin suggested that the lower levels registered in breast cancer patients were due to the lower pineal gland hormone secretion, rather than to an increase in peripheral metabolism. One of the earlier studies considered [38] also found nighttime plasma melatonin levels to be lower in estrogen receptor–positive breast cancer patients compared with estrogen receptor–negative breast cancer patients as well as healthy control women. However, other case–control studies registered a higher daytime serum melatonin concentration in breast cancer patients compared with healthy control subjects [39, 40], thus assuming that there is no correlation between breast cancer risk and the mean daytime nadir and night-time peak plasma concentrations [41]; in addition, the amount of 6-sulfatoxymelatonin excreted in 24-hour urine samples turned to be similar both in women suffering from malignant tumors and in women suffering from benign breast disease [42]. It is difficult to relate such findings to both breast cancer patients and breast cancer–free control subjects, since a series of factors—such as the disease itself, treatment, and/or behavioral changes—which might occur after the diagnosis or before surgery/treatment may actually affect melatonin blood levels. Indeed, several studies found melatonin secretion to be positively or negatively associated with the severity of the disease in terms of tumor size [37], depending on whether the cancer has metastasized [43], as well as whether the patients suffered from primary or secondary tumors or not [36]. It is to be said, however, that such studies were performed on a rather limited sample, and some of them did not even include adequate steps to exclude potential confounding effects relating to age, parity, medication use, or body mass index.

### Melatonin levels and cancer risk

This research has been carried out based on the keywords: “melatonin” AND “secretion” AND “cancer”; 45 articles were sourced. Among them, the most significant epidemiological and retrospective, case–control studies have been selected and discussed.

Epidemiological studies investigated the role of circadian disruption and cancer risk—breast cancer risk in particular—based upon both direct (urinary melatonin levels) and indirect measurements, including sleep duration and shift work. Melatonin production may be closely related to sleep duration, whereas night-shift work is expected to disrupt sleep pattern and thus decrease melatonin levels [44, 45]. To date, epidemiological studies based on direct and indirect melatonin measurements have been carried out upon Western and Asian populations [46] with suggestive results [47, 48]. Breast cancer risk was significantly and inversely associated with urinary melatonin levels (6-sulfatoxymelatonin) in the Nurses’ Health Study II [49], but not in the United Kingdom Guernsey Cohort [50]. Breast cancer risk also resulted to be significantly reduced in association with long sleep duration in Finnish women [51], but not in US women [52, 53]. Results from three different cohort studies [54–56] as well as from two [30, 57] out of three case–control studies [30, 58] also show higher breast cancer risk in women who usually work at evening or in overnight-shifts.

An Italian case–control study nested within the ORDET cohort assessed the concentration of melatonin’s major metabolite, 6-sulfatoxymelatonin (aMT6s), in 178 postmenopausal women with incident invasive breast cancer as well as in 710 matched controls. The multivariate relative risk for women in the highest quartile of total overnight aMT6s output, compared with the lowest, was 0.56 (95 % CI, 0.33–0.97). In this report, overnight urinary aMT6s level and breast cancer risk resulted to be more strongly associated in women who were diagnosed with invasive breast cancer more than 4 years after urine collection (OR 0.34 highest versus lowest quartile, 95 % CI, 0.15–0.75) [59]. In a recent study, the same authors observed a positive correlation between aMT6s and breast cancer risk [60].

Overall, present studies a correlation between night-shift work and breast cancer development in Western countries, as reported by Leonardi et al. [61] in her recent review. Table 1 summarizes the studies investigating the correlation between melatonin levels and cancer risk. Further studies are, however, needed to confirm such a correlation as well as to detect the biomolecular mechanisms which may be involved in the pathogenesis of cancer diagnosed in with night-shift workers.

**Table 1:** Melatonin level and cancer risk

First author	Journal and year	Number of patients	Aim of the study	Results
Sturgeon SR et al. [125]	Cancer Causes Control 2012	48,725 participants in the Women’s Health Initiative Observational Study, among whom 452 adjudicated incident cases of endometrial cancer. 7.5 years of follow-up.	Night-shift work is associated with increased endometrial cancer risk	Indication of reduced risk associated with longer sleep duration, although no statistically significant association was observed.
Wu AH et al. [126]	Carcinogenesis 2008	33,528 women (follow-up 11 years). 525 incident cases of breast cancer	Sleep duration hypothesized to be inversely associated with breast cancer risk	Sleep duration may influence breast cancer risk, possibly via its effect on melatonin levels.
Schernhammer et al. [59]	J Natl Cancer Inst 2008	3,966 eligible postmenopausal women	Low urinary melatonin levels have been associated with an increased risk of breast cancer in premenopausal women.	Results from this prospective study provide evidence for a statistically significant inverse association between melatonin levels, as measured in overnight morning urine, and invasive breast cancer risk in postmenopausal women.
Travis RC et al. [50]	J Natl Cancer Inst 2004	127 patients diagnosed with breast cancer and among 353 control subjects	Experimental data from animals suggest a protective role for the pineal hormone melatonin in the etiology of breast cancer	We found no evidence that the level of melatonin is strongly associated with the risk for breast cancer.
Bartsch C et al. [127]	Clin Chim Acta 1992	24: 8 young men, 7 elderly patients with benign prostatic hyperplasia and nine patients of similar age with primary prostate cancer	Depression of serum melatonin in PC is due to a reduced pineal activity and is not caused by an enhanced metabolic degradation in the liver.	These results imply it is feasible to estimate changes in pineal function of prostate cancer patients by means of non-invasive determination using urinary melatonin and aMT6s.
Bartsch C et al. [36]	Cancer 1991	17 with breast cancer + 4 with untreated benign breast disease	Depression of circulating melatonin in patients with primary breast cancer must be due to a reduced activity of the pineal gland.	The nocturnal melatonin and 6-sulfatoxymelatonin concentrations were significantly depressed in the group of patients with primary breast cancer compared with controls (*P* less than 0.01, *P* less than 0.025). The circadian amplitudes of melatonin and 6-sulfatoxymelatonin were also depressed by 81 % (*P* less than 0.01) and 63 % (*P* less than 0.01).
Bartsch et al. [35]	Cancer 1989	35 with breast cancer + 28 with untreated benign breast disease	Stage-dependent depression of melatonin in patients with primary breast cancer	A 50 % depression of peak and amplitude occurred in the group of patients with primary breast cancer compared with age-matched controls (*P* less than 0.001, *P* less than 0.01). The peak declined with increasing tumor size: 27 % at Stage T1, 53 % at T2 (*P* less than 0.001), and 73 % at T3 (*P* less than 0.05). In contrast, patients with secondary breast cancer, particularly those receiving antiestrogen therapy, had a melatonin peak similar to controls.

### Melatonin, sleep-wake, and mood disturbances in cancer

This research has been carried out based on the keywords: “melatonin” AND “cancer” AND “insomnia” OR “sleep”; 99 articles were sourced. Between these, studies with the greatest number of patients has been selected and discussed.

In cancer patients, depression and its correlated disorders stand for a frequent and severe comorbid condition which may require a special attention [62]. In such patients the prevalence of major depression ranges from 6 to 42 %, whereas based on the 31 reports available, the estimated prevalence rate of depression is 10.8 % [63]. This depends on various cancer-related variables, such as pain and low-performance status, as well as risks for major depression. To avoid the risk of under-diagnosing depression, cancer patients should undergo an accurate psychological assessment, combined with a careful analysis of concomitant physical symptoms, such as anxiety, fatigue, cognitive dysfunction, and sleep disturbances [64–69].

When depression complicates medical conditions, this is usually associated with a substantially reduced quality of life (QOL) [70]. Patients also experience increased symptom burden and greater disability and are less likely to adhere to medical treatments [71].

Although depression is common in cancer patients, this is frequently undetected and untreated, so despite high prevalence of depression, studies on effective pharmacotherapy are relatively scarce and in particular are burdened by a high number of dropouts, due to the side-effects relatingto the use of antidepressants vs placebo [72–75]. The evidence for the efficacy of conventional medication in the treatment of depression, such as tricyclics antidepressants and selective serotonin reuptake inhibitors, is very limited. This could be a consequence of their late onset of action. The use of psychostimulants, which on the contrary grant for a rather rapid onset of action might, therefore, deserve more attention [63].

It is thus rather difficult to clearly determine as to what is the best treatment for major depression disorders (MDD) in cancer patients. It has been assumed that conventional evidence-based treatments for depression in noncancer patients can be applicable [76], although further research is anyway required. Additional studies on how to treat depression are also needed to better understand the role of depression treatments in the improvement of life quality, as it has been proved they may lead to a higher completion of adjuvant therapy and can actually extend lifetime [72, 77, 78].

Literature does not include recent double-blind placebo-controlled studies on the use of melatonin in the treatment of cancer patients’ depression. However, numerous studies actually prove that melatonin is effective in the treatment of major depression in adult [79–81] and elderly patients [21]. In addition the scientific community shows a rising interest on this topic: in April 2012, a protocol article was published in the British Medical Journal to present a clinical trial which should shortly be started. Such a double-blind placebo-controlled trial is to investigate the effect that melatonin performs on breast cancer patients’ depression, anxiety, sleep, and cognitive function disorders [82].

With regard to sleep, the risk of insomnia is usually high in cancer patients [83, 84], yet no large study on the prevalence and nature of cancer patients’ sleep disturbance is available to date. The most interesting study on this topic is that of Davidson et al. [83]. This cross-sectional survey study examined (a) the prevalence of reported sleep problems in the patients registered by six clinics at a regional cancer center, (b) sleep problem prevalence in relation to cancer treatment, and (c) the nature of reported insomnia (type, duration, and associated factors). The most prevalent problems seem to be excessive fatigue (44 % of patients), leg restlessness (41 %), insomnia (31 %), and excessive sleepiness (28 %). The breast clinic registered a high prevalence of insomnia and fatigue. Recent cancer treatment was associated with excessive fatigue and hypersomnolence. Insomnia commonly involved multiple awakenings (76 % of cases) and duration ≥ 6 months (75 % of cases). In most cases (48 %), insomnia onset was reported to coincide with time at which cancer was actually diagnosed (from 6 months pre-diagnosis to 18 months post-diagnosis).

The causes of cancer patients’ sleep disturbance are various, numerous, and pre-existing sleep difficulties often seem to be aggravated by cancer [68].

Cancer itself, including tumors responsible for steroid production and symptoms of tumor invasion (pain, dyspnoea, fatigue, nausea, and pruritus) can also contribute to poor sleep. As a result of chemotherapy, corticosteroid treatment and hormonal fluctuations also affect patients’ sleep and so do medications (narcotics, chemotherapy, neuroleptics, sympathomimetics, sedative/hypnotics, steroids, caffeine/nicotine, antidepressants, and diet supplements) and environmental factors (disturbing light and noise and/or extreme temperature in bedrooms). In addition, the correlation between insomnia and an increased psychological distress due to cancer diagnosis, as well as increased hot flashes caused by menopause, which is often induced by breast cancer treatment, must also be investigated [85]. In cancer patients insomnia may lead to fatigue, mood disturbances, contribute to immunosuppression, affect life quality, and to some extent, may also impact on the course of disease [86] too. The most probable hypothesis about the phenomenology of sleep in breast cancer patients is that the challenges they face may contribute to or cause insomnia, which in turn may exacerbate cancer-associated medical conditions such as pain, psychiatric comorbidities, fatigue, use of opioids (which could contribute to daytime sedation and sleep disorder breathing), stimulating or alerting drugs, napping, and preexisting sleep disturbance [87], thus enhancing a negative feedback loop.

Few pharmacological studies investigating the effect of sleeping pills on cancer patients’ insomnia and other symptoms did not register any significant relevance with respect to the full symptom cluster. Although numerous drugs are currently approved for the treatment of insomnia, to date none of them has been tested for safety or efficacy in cancer patients [84]. No pharmacological treatment has been thus specifically validated for the treatment of cancer cluster symptoms. In a cross-sectional survey carried out in Israel on more than 900 cancer patients, the use of a sleeping pill or a tranquilizer was reported by 25 % and was associated with poorer QOL as well as with increased severity of symptoms like insomnia, fatigue, pain, dyspnoea, and constipation. As a conclusion, authors outlined causal inference is not possible given the cross-sectional design [88]. This reinforces the need for well-controlled clinical trials aiming at detecting the best pharmacological sleep treatments for targeted patients.

With regard to drugs treating insomnia in cancer patients, a study by Casault et al. [89] recently assessed the type and frequency of hypnotic medication among a large sample of randomly selected patients who had been previously treated for cancer. Overall, hypnotic medication resulted to be used by 22.6 % out of the patients’s sample. Factors associated with a larger use of hypnotic medication were older age, greater difficulty to fall asleep, more stressful life events experienced over the past 6 months, higher levels of anxiety, past or current psychological difficulties, poorer role functioning, less severe urinary symptoms, larger use of opioids as well as past or current chemotherapy treatments. In spite of the precautions taken to ensure that the medication was specifically prescribed for sleep disturbance, the registered rate of current use (22.6 %) was very similar to those reported by Davidson et al. [83] (21.5 %) as well as by Paltiel et al. [88] (25.7 %), who had not made any distinction between the use of medication for sleep and the use of anxiolytic medication or tranquilizers for more general purposes. Also to be mentioned, the prevalence rate of hypnotic consumption since the cancer diagnosis was 37 % (for both current and past users). In this study, nearly 80 % out of the participants who were to take drugs were prescribed benzodiazepine (mainly lorazepam and oxazepam), followed by zoplicone (9 %), a non-benzodiazepine hypnotic. Overall, 12.7 % out of the total sample currently used a drug different from the prescribed medication to control sleep disturbance. Moreover, this study showed that three out of four times hypnotics had been prescribed by general practitioners. Based on this, it might be concluded that cancer patients are more inclined to discuss about their sleeping difficulties with their general practitioner, rather than with oncologists, maybe because they feel more at ease, due either to a consolidated relationship of confidence or to the fact that, compared with oncologists, practioners normally perform longer and more informal visits. Such a result is similar to those reported in the epidemiological study by Morin et al. [90], according to which over the previous year 15 % out of the sample (2001 participants from Quebec) had been opting for natural products, 3.8 % had been using over-the-counter medications, whereas 4.1 % of them had been turning to alcohol to treat insomnia. Among cancer patients it might be thus assumed that the consumption rate for substances other than hypnotics appears to be equivalent to the rate generally observed among Quebec’s population. Although sleep experts recommend patients to limit the use of hypnotics to a period of 2–4 weeks, in this study the average duration turned to be close to 5 years. Moreover, a large proportion (78 %) of the sample used it every day. With such an extended and regular usage, tolerance is very likely; this means that for many patients medications had a lower effect over the time, thus leaving them with unrelieved or only partially treated sleep disturbances (e.g., fatigue and mood disturbances).

To date no double-blind placebo-controlled study has been performed to investigate the use of melatonin in the tratment of cancer patients’ sleep disorders.

### Melatonin and anticancer effects

This research has been carried out based on the keywords “melatonin” AND “anticancer” OR “oncostatic”; 59 articles were sourced: 13 about in vitro studies, 28 about animal model studies and 18 about clinical studies. With respect to mood disturbance, the research has been carried out based on the keywords: “melatonin” AND “cancer” AND “depression” OR “mood disturbances”; 99 articles were sourced. Between these, studies with the greatest number of patients has been selected and discussed.

Clinical studies in cancer patients show that [91]:

1. melatonin lowers the toxicity of various chemotherapeutic agents including cisplatin, etoposide, anthracyclines, and 5-fluorouracil;
2. in addition, studies registered a statistically significant reduction in treatment-related adverse events, such as myelosuppression, neurotoxicity, nephrotoxicity, cardiotoxicity and asthenia, which overall result in a decreased mortality rate.

A significantly larger interest for the use of melatonin in cancer treatment was recently raised by the Journal of Pineal Research, publishing the meta-analysis performed by a research group led by Mills et al. [92]. The authors studied 643 cancer patients that did not respond to conventional therapy between 1992 and 2002. Such patients were given melatonin as sole treatment for a variety of different solid tumors including lung, brain, skin, renal, and breast. The effect of large doses of melatonin (10–40 mg/day) was assessed on survival rates after 1 year. The risk of death at 1 year was reduced by 34 %. Effects were consistent depending on melatonin dosage as well as on cancer type. No severe adverse events were reported and the study concluded that substantial reduction in death risk, low side effects, and low costs can actually forecast a larger potential use of melatonin in cancer treatment.

The role of melatonin as an oncostatic drug has been widely documented in in vivo and in vitro experimental investigations, covering a large number of different neoplasias including breast, prostate, colorectal cancer, glioblastoma, leukemia, etc. [93–99]. This definitely clashes with the very limited number of clinical trials aiming at possibly transferring such basic findings into proper clinical protocols [91]. An exhaustive review on anticancer drugs is provided by Grant et al. [18].

### In vitro and in animal model studies

Studies proved melatonin plays a role in the prevention of tumor initiation, promotion, and progression. Melatonin’s oncostatic properties relate to

- its antiproliferative effects [93, 97];
- direct inducing of natural killer cell activity, which enhances immunosurveillance and stimulates cytokine production, such as interleukin 2,6, 12, and interferon gamma [17];
- its ability to increase protein 53, a tumor suppressor protein [100];
- inhibiting linoleic acid uptake via activation of MT1 and MT2 receptors, thereby preventing the formation of the mitogenetic metabolite 1,3-hydroxyoctadecadienoic acid [101];
- its capacity to induce cell differentiation [102];
- its antimetastatic effects [103];
- its anti-angiogenic activity [104];
- its ability to modulate gene expression [105];
- its interaction with estrogen receptors, down-regulating their expression, binding to DNA and transactivation [97, 106, 107];
- its anti aromatase actions [108, 109];
- its modulation of the immune response [110, 111];
- its capacity to decrease telomerase activity [112, 113]; and
- its function as a free radical scavenger [114].

Studies in animal models generally support the hypothesis according to which melatonin can influence the frequency and growth of spontaneous and induced tumors: a pinealectomy can increase tumorigenesis and shorten survival time, whereas administration of melatonin reverses these trends and inhibits tumor growth [115].

### Clinical studies

Melatonin is transmitted through receptors as well as through distinct second-messenger pathways to reduce cellular proliferation and induce cellular differentiation. In addition, independently from receptors, melatonin can also modulate estrogen-dependent pathways and reduce free-radical formation, thus preventing mutation and cellular toxicity. Since melatonin works through a myriad of cell-protecting signaling cascades, this hormone can be suitable for clinical cancer prevention and/or treatment [91].

Wang et al. [116] recent meta-analysis of randomized controlled trials indicates a consistent effect on tumor remission, 1-year survival, and radiochemotherapy-related side effects of adjunct melatonin in a variety of advanced stage cancers. As an adjuvant therapy melatonin led to significantly higher tumor remission, better survival at 1 year, and less radiochemotherapy-related side effects, including thrombocytopenia, neurotoxicity, and fatigue. In many cases cancer had previously resulted to be refractory to standard therapy and thus more suitable for the adjunct use of an untested and unproven therapy like melatonin. The large efficacy as well as the limited number of serious adverse events should actually be of interest to both clinicians and patients. The main limitation is that most [6] trials were performed in the same center [117–122], while only two other studies were performed in different centers [123, 124]. Although the sample size of eight different trials has been enlarged, it is still relatively limited. Since such items may to some extent affect the reliability of the results assessed, authors pointed out international multicentre RCTs with larger sample size are still needed. In the eight studies performed, a once-a-day 20-mg melatonin oral dosage was prescribed. Table 2 summarizes the main studies in which melatonin was administered to cancer patients.

**Table 2:** Clinical studies in which melatonin has been administered to cancer patients

First Author	Journal and year	Number of patients	Diagnosis	Test used for the diagnosis	Dose of melatonin administered to patients	Results
Hansen et al. [82]	BMJ Open 2012	260 (130 × 2)	Breast cancer, depression, anxiety, sleep disturbances and cognitive dysfunction.	Depressive Inventory Mayor, VAS (anxiety), sleep diary, Karolinska Sleepiness Scale, neuropsychological test battery.	6 mg/die	On going
Wang et al. [116]	Cancer Chemother Pharmacol 2012 (review)	761 pts	Solid tumor cancers		20 mg/die	Melatonin as an adjuvant therapy for cancer led to substantial improvements in tumor remission, 1-year survival, and alleviation of radiochemotherapy-related side effects.
Mills et al. [92]	J Pineal Res 2005 (review)	643 pts (between 1992 and 2003)	Solid tumor cancers		Not specified	Melatonin reduced the risk of death at 1 year (relative risk: 0.66, 95 % confidence interval: 0.59–0.73, I2 = 0 %, heterogeneity *P* ≤ 0.56). Effects were consistent across melatonin dose, and type of cancer.
Cerea G et al. [124]	Anticancer Res 2003	30 pts (15 + 15)	Metastatic colorectal cancer		20 mg/die	This preliminary study shows that the efficacy of weekly low-dose CPT-11 in pretreated metastatic colorectal cancer patients may be enhanced by a concomitant daily administration of the pineal hormone MLT
Lissoni P et al. [128]	Eur Urol 1997	14 pts	Metastatic prostate cancer		20 mg/die	A decrease in PSA serum levels greater than 50 % was obtained in 8/14 (57 %) patients, a survival longer than 1 year was achieved in 9/14 (64 %) patients. The concomitant administration of the pineal hormone MLT may overcome clinical resistance to LHRH analogs and improve clinical conditions in metastatic prostatic cancer patients.
Lissoni P et al. [121]	Oncology 1996	30 pts (15 + 15)	Brain glioblastoma		20 mg/die	Both the survival curve and the survival % at 1 year were significantly higher in patients treated with RT plus MLT than in those receiving RT alone (6/14 vs. 1/16).
Barni S et al. [129]	Oncology 1995	50	Metastatic colorectal cancer		40 mg/die	This study suggests low-dose subcutaneous IL-2 plus melatonin may be effective as a second-line therapy to induce tumor regression and to prolong % survival at 1 year in metastatic colorectal cancer patients progressing under 5-FU and folates.
Lissoni P et al. [130]	Br J Cancer 1995	14 pts	Metastatic breast cancer		20 mg/die	A partial response was achieved in 4/14 (28.5 %) patients (median duration 8 months. The concomitant administration of the pineal hormone MLT may induce objective tumor regressions in metastatic breast cancer patients refractory to TMX alone.
Lissoni P et al. [131]	Oncol Rep 1995	40 pts (20 + 20)	Breast cancer		20 mg/die	Partial response rate was significantly higher in patients treated with TMX and MLT than in those, who received TMX alone (7/19 vs 2/21, *P* < 0.05). Moreover, the survival % at 1 year was significantly higher in patients treated with TMX plus MLT than in those treated with TMX alone (12/19 vs 5/21, P < 0.01).
Lissoni P et al. [132]	Cancer 1994	50 (25 × 2)	Brain metastases		20 mg/die	The pineal hormone melatonin may be able to improve the survival time and the quality of life in patients with brain metastases due to solid tumors.
Aldeghi R et al. [133]	Eur J Cancer 1994	14 pts	Hepatocellular carcinoma		50 mg/die	Objective tumor regressions were obtained in 5/14 (36 %) patients.

### Toxicity studies of melatonin

The vast majority of studies document the very low toxicity of melatonin over a wide range of doses, even in up to 20-mg dosages, as reported by Sánchez-Barceló et al. [91], where a number of clinical trials have been reviewed to assess the therapeutic usefulness of melatonin in different medical fields.

## References

[1] Claustrat B, Brun J, Chazot G (2005). The basic physiology and pathophysiology of melatonin. Sleep Med Rev.

[2] Bubenik GA (2002). Gastrointestinal melatonin: localization, function, and clinical relevance. Dig Dis Sci.

[3] Hardeland R (2005). Antioxidative protection by melatonin: multiplicity of mechanisms from radical detoxification to radical avoidance. Endocrine.

[4] Jan JE, Reiter RJ, Wasdell MB, Bax M (2009). The role of the thalamus in sleep, pineal melatonin production, and circadian rhythm sleep disorders. J Pineal Res.

[5] Sack RL, Auckley D, Auger RR (2007). Circadian rhythm sleep disorders: part I, basic principles, shift work and jet lag disorders. An American Academy of Sleep Medicine review. Sleep.

[6] Schulz P, Steimer T (2009). Neurobiology of circadian systems. CNS Drugs.

[7] Tzischinsky O, Shlitner A, Lavie P (1993). The association between the nocturnal sleep gate and nocturnal onset of urinary 6- sulphatoxymelatonin. J Biol Rhythms.

[8] Zhdanova IV, Wurtman RJ (1997). Efficacy of melatonin as a sleep-promoting agent. J Biol Rhythms.

[9] Brzezinski A (1997). Melatonin in humans. N Engl J Med.

[10] Acil M, Basgul E, Celiker V, Karagöz AH, Demir B, Aypar U (2004). Perioperative effects of melatonin and midazolam premedication on sedation, orientation, anxiety scores and psychomotor performance. Eur J Anaesthesiol.

[11] Naguib M, Samarkandi AH (1999). Premedication with melatonin: a doubleblind, placebo-controlled comparison with midazolam. Br J Anaesth.

[12] Caumo W, Torres F, Moreira NL (2007). The clinical impact of preoperative melatonin on postoperative outcomes in patients undergoing abdominal hysterectomy. Anesth Analg.

[13] Srinivasan V, Pandi-Perumal SR, Spence DW (2010). Potential use of melatonergic drugs in analgesia: mechanisms of action. Brain Res Bull.

[14] Cagnacci A, Cannoletta M, Renzi A, Baldassari F, Arangino S, Volpe A (2005). Prolonged melatonin administration decreases nocturnal blood pressure in women. Am J Hypertens.

[15] Kitajima T, Kanbayashi T, Saitoh Y (2001). The effects of oral melatonin on the autonomic function in healthy subjects. Psychiatry Clin Neurosci.

[16] Maestroni GJ (2001). The immunotherapeutic potential of melatonin. Expert Opin Investig Drugs.

[17] Vijayalaxmi S, Reiter RJ, Tan DX, Herman TS, Thomas CR Jr (2002) Melatonin: from basic research to cancer treatment clinics. J Clin Oncol 20: 2575–2601 10.1200/JCO.2002.11.004 12011138

[18] Grant SG, Melan MA, Latimer JJ, Witt-Enderby PA (2009). Melatonin and breast cancer: cellular mechanisms, clinical studies and future perspectives. Expert Rev Mol Med.

[19] Reiter RJ (2004). Mechanisms of cancer inhibition by melatonin. J Pineal Res.

[20] Rahman SA, Kayumov L, Shapiro CM (2010). Antidepressant action of melatonin in the treatment of Delayed Sleep Phase Syndrome. Sleep Med.

[21] Rondanelli M, Opizzi A, Monteferrario F, Antoniello N, Manni R, Klersy C (2011). The effect of melatonin, magnesium, and zinc on primary insomnia in long-term care facility residents in Italy: a double-blind, placebo-controlled clinical trial. J Am Geriatr Soc.

[22] Srinivasan V, Pandi-Perumal SR, Trakht I (2009). Pathophysiology of depression: role of sleep and the melatonergic system. Psychiatry Res.

[23] Furio AM, Brusco LI, Cardinali DP (2007). Possible therapeutic value of melatonin in mild cognitive impairment: a retrospective study. J Pineal Res.

[24] Rondanelli M, Opizzi A, Faliva M (2012). Effects of a diet integration with an oily emulsion of DHA-phospholipids containing melatonin and tryptophan in elderly patients suffering from mild cognitive impairment. Nutr Neurosci.

[25] Srinivasan V, Smits M, Spence W (2006). Melatonin in mood disorders. World J Biol Psychiatry.

[26] Garzòn C, Guerrero JM, Aramburu O, Guzmán T (2009). Effect of melatonin administration on sleep, behavioral disorders and hypnotic drug discontinuation in theelderly: a randomized, double-blind, placebo-controlled study. Aging Clin Exp Res.

[27] Serfaty MA, Osborne D, Buszewicz MJ, Blizard R, Raven PW (2010). A randomized doubleblind placebo-controlled trial of treatment as usual plus exogenous slow-release melatonin (6 mg) or placebo for sleep disturbance and depressed mood. Int Clin Psychopharmacol.

[28] Spadoni G, Bedini A, Rivara S, Mor M (2010). Melatonin receptor agonists: new options for insomnia and depression treatment. CNS Neurosci Ther.

[29] Lissoni P (2002). Is there a role for melatonin in supportive care?. Support Care Cancer.

[30] Davis S, Mirick DK, Stevens RG (2001). Night-shift work, light at night, and risk of breast cancer. J Natl Cancer Inst.

[31] Reiter RJ, Tan DX, Korkmaz A (2007). Light at night, chronodisruption, melatonin suppression, and cancer risk: a review. Crit Rev Oncog.

[32] Straif K, Baan R, Grosse Y (2007). Carcinogenicity of shift-work, painting, and fire-fighting. Lancet Oncol.

[33] Costa G, Haus E, Stevens R (2010). Shiftwork and cancer: considerations on rationale, mechanisms, and epidemiology. Scand J Work Environ Health.

[34] Bartsch C, Bartsch H, Jain AK, Laumas KR, Wetterberg L (1981). Urinary melatonin levels in human breast cancer patients. J Neural Transm.

[35] Bartsch C, Bartsch H, Fuchs U, Lippert TH, Bellmann O, Gupta D (1989). Stage-dependent depression of melatonin in patients with primary breastcancer. Correlation with prolactin, thyroid stimulating hormone, and steroid receptors. Cancer.

[36] Bartsch C, Bartsch H, Bellmann O, Lippert TH (1991). Depression of serum melatonin in patients with primary breast cancer is not due to an increased peripheral metabolism. Cancer.

[37] Bartsch C, Bartsch H, Karenovics A, Franz H, Peiker G, Mecke D (1997). Nocturnal urinary 6-sulphatoxymelatonin excretion is decreased in primary breast cancer patients compared to age-matched controls and shows negative correlation with tumor-size. J Pineal Res.

[38] Tamarkin L, Danforth D, Lichter A (1982). Decreased nocturnal plasma melatonin peak in patients with estrogen receptor positive breast cancer. Science.

[39] Lissoni P, Bastone A, Sala R (1987). The clinical significance of melatonin serum determination in oncological patients and its correlations with GH and PRL blood levels. Eur J Cancer Clin Oncol.

[40] Lissoni P, Crispino S, Barni S (1990). Pineal gland and tumor cell kinetics: serum levels of melatonin in relation to Ki-67 labeling rate in breast cancer. Oncology.

[41] Danforth DN, Tamarkin L, Mulvihill JJ, Bagley CS, Lippman ME (1985). Plasma melatonin and the hormone-dependency of human breast cancer. J Clin Oncol.

[42] Skene DJ, Bojkowski CJ, Currie JE, Wright J, Boulter PS, Arendt J (1990). 6-Sulphatoxymelatonin production in breast cancer patients. J Pineal Res.

[43] Falkson G, Falkson HC, Steyn ME, Rapoport BL, Meyer BJ (1990). Plasma melatonin in patients with breast cancer. Oncology.

[44] Erren TC (2002). Does light cause internal cancers? The problem and challenge of an ubiquitous exposure. Neuro Endocrinol Lett.

[45] Arendt J (1998). Melatonin and the pineal gland: influence on mammalian seasonal and circadian physiology. Rev Reprod.

[46] Wu AH, Stanczyk FZ, Wang R, Koh WP, Yuan JM, Yu MC (2012). Sleep duration, spot urinary 6-sulfatoxymelatonin levels and risk of breast cancer among Chinese women in Singapore. Int J Cancer.

[47] Megdal SP, Kroenke CH, Laden F, Pukkala E, Schernhammer ES (2005). Night work and breast cancer risk: a systematic review and meta-analysis. Eur J Cancer.

[48] Stevens RG (2006). Artificial lighting in the industrialized world: circadian disruption and breast cancer. Cancer Causes Control.

[49] Schernhammer ES, Hankinson SE (2005). Urinary melatonin levels and breast cancer risk. J Natl Cancer Inst.

[50] Travis RC, Allen DS, Fentiman IS, Key TJ (2004). Melatonin and breast cancer: a prospective study. J Natl Cancer Inst.

[51] Verkasalo PK, Lillberg K, Stevens RG (2005). Sleep duration and breast cancer: a prospective cohort study. Cancer Res.

[52] McElroy JA, Newcomb PA, Titus-Ernstoff L, Trentham-Dietz A, Hampton JM, Egan KM (2006). Duration of sleep and breast cancer risk in a large population-based case-control study. J Sleep Res.

[53] Pinheiro SP, Schernhammer ES, Tworoger SS, Michels KB (2006). A prospective study on habitual duration of sleep and incidence of breast cancer in a large cohort of women. Cancer Res.

[54] Lie JA, Roessink J, Kjaerheim K (2006). Breast cancer and night work among Norwegian nurses. Cancer Causes Control.

[55] Schernhammer ES, Kroenke CH, Laden F, Hankinson SE (2006). Night work and risk of breast cancer. Epidemiology.

[56] Schernhammer ES, Laden F, Speizer FE (2001). Rotating night-shifts and risk of breast cancer in women participating in the nurses’ health study. J Natl Cancer Inst.

[57] Hansen J (2001). Light at night, shiftwork, and breast cancer risk. J Natl Cancer Inst.

[58] O’Leary ES, Schoenfeld ER, Stevens RG, Kabat GC, Henderson K, Grimson R (2006). Shift work, light at night, and breast cancer on Long Island, New York. Am J Epidemiol.

[59] Schernhammer ES, Berrino F, Krogh V (2008). Urinary 6-sulfatoxymelatonin levels and risk of breast cancer in postmenopausal women. J Natl Cancer Inst.

[60] Schernhammer ES, Berrino F, Krogh V, Secreto G, Micheli A, Venturelli E (2010). Urinary 6-Sulphatoxymelatonin levels and risk of breast cancer in postmenopausal women: the ORDET cohort. Cancer Epidemiol Biomarkers Prev.

[61] Leonardi GC, Rapisarda V, Marconi A (2012). Correlation of the risk of breast cancer and disruption of the circadian rhythm (Review). Oncol Rep.

[62] Uchitomi Y (2001). Depression in cancer patients. Nihon Rinsho.

[63] Ng CG, Boks MP, Zainal NZ, de Wit NJ (2011). The prevalence and pharmacotherapy of depression in cancer patients. J Affect Disord.

[64] Fann JR, Thomas-Rich AM, Katon WJ, Cowley D (2008). Major depression after breast cancer: a review of epidemiology and treatment. Gen Hosp Psychiatry.

[65] Burgess C, Cornelius V, Love S, Graham J, Richards M, Ramirez A (2005). Depression and anxiety in women with early breast cancer: five year observational cohort study. BMJ.

[66] Otte JL, Carpenter JS, Russell KM, Bigatti S, Champion VL (2010). Prevalence, severity, and correlates of sleep-wake disturbances in long-term breast cancer survivors. J Pain Symptom Manage.

[67] Debess J, Riis JO, Pedersen L, Ewertz M (2009). Cognitive function and quality of life after surgery for early breast cancer in North Jutland, Denmark. Acta Oncol.

[68] Savard J, Simard S, Blanchet J, Ivers H, Morin CM (2001). Prevalence, clinical characteristics, and risk factors for insomnia in the context of breast cancer. Sleep.

[69] Massie MJ (2004). Prevalence of depression in patients with cancer. J Natl Cancer Inst Monogr.

[70] Moussavi S, Chatterji S, Verdes E, Tandon A, Patel V, Ustun B (2007). Depression, chronic diseases, and decrements in health: results from the World Health Surveys. Lancet.

[71] Katon W, Sullivan MD (1990). Depression and chronic medical illness. J Clin Psychiatry.

[72] Navari RM, Brenner MC, Wilson MN (2008). Treatment of depressive symptoms in patients with early stage breast cancer undergoing adjuvant therapy. Breast Cancer Res Treat.

[73] Roscoe JA, Morrow GR, Hickok JT (2005). Effect of paroxetine hydrochloride (Paxil) on fatigue and depression in breast cancer patients receiving chemotherapy. Breast Cancer Res Treat.

[74] Musselman DL, Somerset WI, Guo Y (2006). A double-blind, multicenter, parallel-group study of paroxetine, desipramine, or placebo in breast cancer patients (stages I, II, III, and IV) with major depression. J Clin Psychiatry.

[75] Morrow GR, Hickok JT, Roscoe JA (2003). Differential effects of paroxetine on fatigue and depression: a randomized, double-blind trial from the University of Rochester Cancer Center Community Clinical Oncology Program. J Clin Oncol.

[76] Geddes J, Butler R (2002). Depressive disorders. Clin Evid.

[77] Somerset W, Stout SC, Miller AH, Musselman D (2004). Breast cancer and depression. Oncology (Williston Park).

[78] Giese-Davis J, Collie K, Rancourt KM, Neri E, Kraemer HC, Spiegel D (2011). Decrease in depression symptoms is associated to longer survival in patients with metastatic breast cancer: a secondary analysis. J Clin Oncol.

[79] Lewy AJ, Bauer VK, Cutler NL, Sack RL (1998). Melatonin treatment of winter depression: a pilot study. Psychiatry Res.

[80] Lewy AJ, Emens J, Jackman A, Yuhas K (2006). Circadian uses of melatonin in humans. Chronobiol Int.

[81] Dolberg OT, Hirschmann S, Grunhaus L (1998). Melatonin for the treatment of sleep disturbances in major depressive disorder. Am J Psychiatry.

[82] Hansen MV, Madsen MT, Hageman I (2012). The effect of MELatOnin on Depression, anxietY, cognitive function and sleep disturbances in patients with breast cancer. The MELODY trial: protocol for a randomised placebo-controlled double-blinded trial. BMJ Open.

[83] Davidson JR, MacLean AW, Brundage MD, Schulze K (2002). Sleep disturbance in cancer patients. Soc Sci Med.

[84] Savard J, Morin CM (2001). Insomnia in the context of cancer: a review of a neglected problem. J Clin Oncol.

[85] O’Donnell JF (2004). Insomnia in cancer patients. Clin Cornerstone.

[86] Bardwell WA, Profant J, Casden DR (2008). The relative importance of specific risk factors for insomnia in women treated for early-stage breast cancer. Psychooncology.

[87] Fiorentino L, Ancoli-Israel S (2007). Sleep dysfunction in patients with cancer. Curr Treat Options Neurol.

[88] Paltiel O, Marzec-Boguslawska A, Soskolne V (2004). Use of tranquilizers and sleeping pills among cancer patients is associated to a poorer quality of life. Qual Life Res.

[89] Casault L, Savard J, Ivers H, Savard MH, Simard S (2012). Utilization of hypnotic medication in the context of cancer: predictors and frequency of use. Support Care Cancer.

[90] Morin CM, LeBlanc M, Daley M, Gregoire JP, Merette C (2006). Epidemiology of insomnia: prevalence, self-help treatments, consultations, and determinants of help-seeking behaviors. Sleep Med.

[91] Sánchez-Barceló EJ, Mediavilla MD, Tan DX, Reiter RJ (2010). Clinical uses of melatonin: evaluation of human trials. Curr Med Chem.

[92] Mills E, Wu P, Seely D, Guyatt G (2005). Melatonin in the treatment of cancer: a systematic review of randomized controlled trials and meta-analysis. J Pineal Res.

[93] Cos S, Sanchez-Barcelo EJ (2000). Melatonin and mammary pathological growth. Front Neuroendocrinol.

[94] Korkmaz A, Sanchez-Barcelo EJ, Tan DX, Reiter RJ (2009). Role of melatonin in the epigenetic regulation of breast cancer. Breast Cancer Res Treat.

[95] Jung B, Ahmad N (2006). Melatonin in cancer management: progress and promise. Cancer Res.

[96] Witt-Enderby PA, Radio NM, Doctor JS, Davis VL (2006). Therapeutic treatments potentially mediated by melatonin receptors: potential clinical uses in the prevention of osteoporosis, cancer and as an adjuvant therapy. J Pineal Res.

[97] Sánchez-Barceló EJ, Cos S, Mediavilla D, Martínez-Campa C, González A, Alonso-González C (2005). Melatonin-estrogen interactions in breast cancer. J Pineal Res.

[98] Cos S, González A, Martínez-Campa C, Mediavilla MD, Alonso-González C, Sánchez-Barceló EJ (2006). Estrogen-signaling pathway: a link between breast cancer and melatonin oncostatic actions. Cancer Detect Prev.

[99] Cos S, Garcia-Bolado A, Sánchez-Barceló EJ (2001). Direct antiproliferative effects of melatonin on two metastatic cell sublines of mouse melanoma (B16BL6 and PG19). Melanoma Res.

[100] Mediavilla MD, Cos S, Sánchez-Barceló EJ (1999). Melatonin increases p53 and p21WAF1 expression in MCF-7 human breast cancer cells in vitro. Life Sci.

[101] Martin-Renedo J, Mauriz JL, Jorquera F, Ruiz-Andres O, Gonzalez P, Gonzalez-Gallego J (2008). Melatonin induces cell cycle arrest and apoptosis in hepatocarcinoma HepG2 cell line. J Pineal Res.

[102] Radio NM, Doctor JS, Witt-Enderby PA (2006). Melatonin enhances alkaline phosphatase activity in differentiating human adult mesenchymal stem cells grown in osteogenic medium via MT2 melatonin receptors and the MEK/ERK (1/2) signaling cascade. J Pineal Res.

[103] Cos S, Fernández R, Güézmes A, Sánchez-Barceló EJ (1998). Influence of melatonin on invasive and metastatic properties of MCF-7 human breast cancer cells. Cancer Res.

[104] Lissoni P, Rovelli F, Malugani F, Bucovec R, Conti A, Maestroni GJ (2001). Anti-angiogenic activity of melatonin in advanced cancer patients. Neuro Endocrinol Lett.

[105] Mediavilla MD, Güezmez A, Ramos S, Kothari L, Garijo F, Sánchez Barceló EJ (1997). Effects of melatonin on mammary gland lesions in transgenic mice overexpressing N-ras proto-oncogene. J Pineal Res.

[106] Rato AG, Pedrero JG, Martinez MA, del Rio B, Lazo PS, Ramos S (1999). Melatonin blocks the activation of estrogen receptor for DNA binding. FASEB J.

[107] del Río B, García Pedrero JM, Martínez-Campa C, Zuazua P, Lazo PS, Ramos S (2004). Melatonin, an endogenousspecific inhibitor of estrogen receptor alpha via calmodulin. J Biol Chem.

[108] Gonzalez A, Cos S, Martinez-Campa C, Alonso-Gonzalez C, Sanchez-Mateos S, Mediavilla MD (2008). Selective estrogen enzyme modulator actions of melatonin in human breast cancer cells. J Pineal Res.

[109] Cos S, González A, Güezmes A (2006). Melatonin inhibits the growth of DMBA-induced mammary tumors by decreasing the local biosynthesis of estrogens through the modulation of aromatase activity. Int J Cancer.

[110] Carrillo-Vico A, Reiter RJ, Lardone PJ (2006). The modulatory role of melatonin on immune responsiveness. Curr Opin Investig Drugs.

[111] Carrillo-Vico A, García-Mauriño S, Calvo JR, Guerrero JM (2003). Melatonin counteracts the inhibitory effect of PGE2 on IL-2 production in human lymphocytes via its mt1 membrane receptor. FASEB J.

[112] Leon-Blanco MM, Guerrero JM, Reiter RJ, Calvo JR, Pozo D (2003). Melatonin inhibits telomerase activity in the MCF-7 tumor cell line both in vivo and in vitro. J Pineal Res.

[113] Martínez-Campa CM, Alonso-González C, Mediavilla MD, Cos S, González A, Sanchez-Barcelo EJ (2008). Melatonin downregulates hTERT expression induced by either natural estrogens (17beta-estradiol) or metalloestrogens (cadmium) in MCF-7 human breast cancer cells. Cancer Lett.

[114] Tan DX, Reiter RJ, Manchester LC (2002). Chemical and physical properties and potential mechanisms: melatonin as a broad spectrum antioxidant and free radical scavenger. Curr Topics Med Chem.

[115] Tamarkin L, Cohen M, Roselle D, Reichert C, Lippman M, Chabner B (1981). Melatonin inhibition and pinealectomy enhancement of 7,12- dimethylbenz(a)anthracene-induced mammary tumors in the rat. Cancer Res.

[116] Wang YM, Jin BZ, Ai F (2012). The efficacy and safety of melatonin in concurrent chemotherapy or radiotherapy for solid tumors: a meta-analysis of randomized controlled trials. Cancer Chemother Pharmacol.

[117] Lissoni P, Tancini G, Barni S (1997). Treatment of cancer chemotherapy-induced toxicity with the pineal hormone melatonin. Support Care Cancer.

[118] Lissoni P, Barni S, Mandala M (1999). Decreased toxicity and increased eYcacy of cancer chemotherapy using the pineal hormone melatonin in metastatic solid tumor patients with poor clinical status. Eur J Cancer.

[119] Lissoni P, Paolorossi F, Ardizzoia A (1997). A randomized study of chemotherapy with cisplatin plus etoposide versus chemoendocrine therapy with cisplatin, etoposide and the pineal hormone melatonin as a Wrst-line treatment of advanced non-small cell lung cancer patients in a poor clinical state. J Pineal Res.

[120] Lissoni P (2007). Biochemotherapy with immunomodulating pineal hormones other than melatonin: 5-methoxytryptamine as a new oncostatic pineal agent. Pathol Biol (Paris).

[121] Lissoni P, Meregalli S, Nosetto L (1996). Increased survival time in brain glioblastomas by a radioneuroendocrine strategy with radiotherapy plus melatonin compared to radiotherapy alone. Oncology.

[122] Lissoni P, Chilelli M, Villa S, Cerizza L, Tancini G (2003). Five years survival in metastatic non small cell lung cancer patients treated with chemotherapy alone or chemotherapy and melatonin: a randomized trial. J Pineal Res.

[123] Yan J, Shen F, Wang K, Wu M (2001). Co-antitumor effect andhepatic protection of melatonin on advanced primary liver cancertreated by transcatheter arterial chemoembolization. Acad J Sec Mil Med Univ.

[124] Cerea G, Vaghi M, Ardizzoia A (2003). Biomodulation of cancer chemotherapy for metastatic colorectal cancer: a randomized study of weekly low-dose irinotecan alone versus irinotecan plus the oncostatic pineal hormone melatonin in metastatic colorectal cancer patients progressing on 5-Xuorouracil-containing combinations. Anticancer Res.

[125] Sturgeon SR, Luisi N, Balasubramanian R, Reeves KW (2012) Sleep duration and endometrial cancer risk. Cancer Causes Control 23:547–553 10.1007/s10552-012-9912-2 22362059

[126] Wu AH, Wang R, Koh WP, Stanczyk FZ, Lee HP, Yu MC (2008) Sleep duration, melatonin and breast cancer among Chinese women in Singapore. Carcinogenesis 29:1244–1248 10.1093/carcin/bgn100 PMC2574570 18448486

[127] Bartsch C, Bartsch H, Schmidt A, Ilg S, Bichler KH, Flüchter SH (1992) Melatonin and 6-sulfatoxymelatonin circadian rhythms in serum and urine of primary prostate cancer patients: evidence for reduced pineal activity and relevance of urinary determinations. Clin Chim Acta 209:153–167 10.1016/0009-8981(92)90164-l 1395046

[128] Lissoni P, Cazzaniga M, Tancini G, Scardino E, Musci R, Barni S, Maffezzini M, Meroni T, Rocco F, Conti A, Maestroni G (1997) Reversal of clinical resistance to LHRH analogue in metastatic prostate cancer by the pineal hormone melatonin: efficacy of LHRH analogue plus melatonin in patients progressing on LHRH analogue alone. Eur Urol 31(2):178–181 10.1159/000474446 9076462

[129] Barni S, Lissoni P, Cazzaniga M, Ardizzoia A, Meregalli S, Fossati V, Fumagalli L, Brivio F, Tancini G (1995) A randomized study of low-dose subcutaneous interleukin-2 plus melatonin versus supportive care alone in metastatic colorectal cancer patients progressing under 5-fluorouracil and folates. Oncology 52:243–245 10.1159/000227465 7715908

[130] Lissoni P, Barni S, Meregalli S, Fossati V, Cazzaniga M, Esposti D, Tancini G (1995) Modulation of cancer endocrine therapy by melatonin: a phase II study of tamoxifen plus melatonin in metastatic breast cancer patients progressing under tamoxifen alone. Br J Cancer 71:854–856 10.1038/bjc.1995.164 PMC2033724 7710954

[131] Lissoni P, Ardizzoia A, Barni S, Paolorossi F, Tancini G, Meregalli S, Esposti D, Zubelewicz B, Braczowski R (1995) A randomized study of tamoxifen alone versus tamoxifen plus melatonin in estrogen receptor-negative heavily pretreated metastatic breast-cancer patients. Oncol Rep 2:871–873 10.3892/or.2.5.871 21597833

[132] Lissoni P, Barni S, Ardizzoia A, Tancini G, Conti A, Maestroni G (1994) A randomized study with the pineal hormone melatonin versus supportive care alone in patients with brain metastases due to solid neoplasms. Cancer 73:699–701 10.1002/1097-0142(19940201)73:3<699::aid-cncr2820730332>3.0.co;2-l 8299092

[133] Aldeghi R, Lissoni P, Barni S, Ardizzoia A, Tancini G, Piperno A, Pozzi M, Ricci G, Conti A, Maestroni GJ (1194) Low-dose interleukin-2 subcutaneous immunotherapy in association with the pineal hormone melatonin as a first-line therapy in locally advanced or metastatic hepatocellular carcinoma. Eur J Cancer 30A:167–170 10.1016/0959-8049(94)90080-9 8155391

